# Glucocorticoid-Induced Exacerbation of Mycobacterial Infection Is Associated With a Reduced Phagocytic Capacity of Macrophages

**DOI:** 10.3389/fimmu.2021.618569

**Published:** 2021-05-11

**Authors:** Yufei Xie, Jiajun Xie, Annemarie H. Meijer, Marcel J. M. Schaaf

**Affiliations:** Institute of Biology, Leiden University, Leiden, Netherlands

**Keywords:** glucocorticoids, *Mycobacterium marinum*, macrophage, phagocytosis, zebrafish, tuberculosis

## Abstract

Glucocorticoids are effective drugs for treating immune-related diseases, but prolonged therapy is associated with an increased risk of various infectious diseases, including tuberculosis. In this study, we have used a larval zebrafish model for tuberculosis, based on *Mycobacterium marinum* (*Mm*) infection, to study the effect of glucocorticoids. Our results show that the synthetic glucocorticoid beclomethasone increases the bacterial burden and the dissemination of a systemic *Mm* infection. The exacerbated *Mm* infection was associated with a decreased phagocytic activity of macrophages, higher percentages of extracellular bacteria, and a reduced rate of infected cell death, whereas the bactericidal capacity of the macrophages was not affected. The inhibited phagocytic capacity of macrophages was associated with suppression of the transcription of genes involved in phagocytosis in these cells. The decreased bacterial phagocytosis by macrophages was not specific for *Mm*, since it was also observed upon infection with *Salmonella* Typhimurium. In conclusion, our results show that glucocorticoids inhibit the phagocytic activity of macrophages, which may increase the severity of bacterial infections like tuberculosis.

## Highlights

Using a zebrafish tuberculosis model, we show that glucocorticoids increase the severity of the bacterial infection, and decrease the phagocytosis by macrophages. This may explain the glucocorticoid-induced increase in susceptibility to tuberculosis in humans.

## Introduction

Glucocorticoids (GCs) are a class of steroid hormones that are secreted upon stress. The main endogenous GC in our body, cortisol, helps our bodies adapt to stressful situations and for this purpose it regulates a wide variety of systems, like the immune, metabolic, reproductive, cardiovascular and central nervous system. These effects are mediated by an intracellular receptor, the glucocorticoid receptor (GR), which acts as a ligand-activated transcription factor. Synthetic GCs are widely prescribed to treat various immune-related diseases due to their potent suppressive effects on the immune system. However, prolonged therapy with these pleiotropic steroids evokes severe side effects, such as osteoporosis and diabetes mellitus (1, 2). Importantly, the therapeutic immunosuppressive effect of GCs may lead to infectious complications because of the compromised immune system (3–5). Similarly, after chronic stress an increased susceptibility to infectious diseases has been observed, due to the high circulating levels of cortisol. In order to better understand these complex effects of GCs, more research is required into how GCs influence the susceptibility to infections and the course of infectious diseases.

Tuberculosis (TB) is the most prevalent bacterial infectious disease in the world, caused by the pathogen *Mycobacterium tuberculosis* (*Mtb*). Despite the efforts made to reach the “End TB Strategy” of the World Health Organization, *Mtb* still infects approximately one-quarter of the world’s population and caused an estimated 1.5 million deaths in 2018, which makes it one of the top 10 causes of death globally (6, 7). The major characteristic of *Mtb* infection is the formation of granulomas containing infected and non-infected immune cells (8). Most *Mtb-*infected people develop a latent, noncontagious infection and do not show any symptoms, with the bacteria remaining inactive, while contained within granulomas (9, 10). About 5-10% of the carriers develop a clinically active TB disease associated with a loss of granuloma integrity (9, 11). Among those TB patients, the majority manifest a lung infection and around 20% shows infection in other organs like the central nervous system, pleura, urogenital tracts, bones and joints, and lymph nodes (12). Antibiotics are currently the mainstay for TB treatment, but since antibiotic resistance is rising and an effective vaccine against latent or reactivated TB is still lacking, alternative therapies to control TB are needed (13).

GCs are known to modulate the pathogenesis of TB, but their effects are highly complicated. The use of GCs is considered as a risk factor for TB. Patients who are being treated with GCs have an approximately 5-fold increased risk for developing new TB (14), and treatment with a moderate or high dose of GCs is associated with an increased risk of activation of latent TB (15–17). Consequently, a tuberculin skin test (TST) for screening latent TB is recommended before starting GC therapy (14). Moreover, chronic stress which is associated with increased circulating levels of the endogenous GC cortisol, has been shown to be associated with a higher incidence of TB (18).

Despite the generally detrimental effects of GCs on TB susceptibility and progression, certain types of TB patients are treated with GCs. Chronic TB patients may require GCs for treatment of other disorders, and it has been shown that adjunctive GC therapy may have beneficial effects. Traditionally, adjunctive GC with standard anti-TB therapy has been used for prevention of inflammatory complications in patients with tuberculous meningitis, pericarditis, and pleurisy (19–22). It has been reported that adjunctive GC therapy could improve the probability of survival in tuberculous meningitis and pericarditis (23–26). In case of pulmonary TB, the most common form of TB, adjunctive GC therapy is recommended in advanced tuberculosis since broad and significant clinical benefits have been demonstrated (27, 28).

Although GCs are being used for adjunctive therapy, the beneficial effects of GC treatment are still under debate. For tuberculous pleurisy TB, the efficacy of GCs is still controversial and for meningitis and pericarditis, information on the GC effects is still incomplete (22, 26, 29, 30). A review regarding clinical trials for pulmonary TB showed that, although adjunctive GC therapy appears to have short-term benefits, it is not maintained in the long-term (31). An explanation for the complexity of the effects of GC therapy in TB has been offered by Tobin et al. (2012). They showed that patients suffer from TB as a result of either a failed or an excessive immune response to the mycobacterial infection, and that only the subset of TB meningitis patients with an excessive response, showing a hyperinflammatory phenotype (in their study as a result of a polymorphism in the *LTA4H* gene), benefited from adjunctive GC therapy. It was suggested that GCs may also be beneficial for similar subgroups of patients suffering from other forms of TB (32).

The complex interplay between GC actions and TB underscores the need for a better understanding of the effects of GCs on mycobacterial infection. In the present study we have studied these effects using *Mycobacterium marinum* (*Mm*) infection in zebrafish as a model system. *Mm* is a species closely related to *Mtb* that can infect zebrafish and other cold-blooded animals naturally, causing a TB-like disease (33). Infection of zebrafish larvae with *Mm* provides an animal model system that mimics hallmark aspects of *Mtb* infection in humans and is widely used for research into mechanisms underlying the course of this disease (34–36). Like *Mtb, Mm* is able to survive and replicate within macrophages and, in later stages of infection, induces the formation of granulomas (37). The transparency of zebrafish at early life stages makes it possible to perform non-invasive long-term live imaging, which has been used to reveal the earliest stages of granuloma formation (38). In addition, the availability of different transgenic and mutant zebrafish lines and the efficient application of molecular techniques allow us to exploit this zebrafish *Mm* infection model optimally to study both the host factors and bacterial factors involved in mycobacterial infection processes (33, 34, 39). For example, zebrafish studies revealed that infected macrophages can detach from a granuloma and facilitate dissemination to new locations (38). Moreover, the study of an *lta4h* mutant zebrafish line showed that the polymorphism in the *LTA4H* gene is associated with the susceptibility to mycobacterial diseases and the response to adjunctive GC therapy in human, representing a prime example of translational research (32, 40).

The zebrafish has proven to be a suitable model for studying the effects of GCs, since the GC signaling pathway is very well conserved between zebrafish and humans. Both humans and zebrafish have a single gene encoding the GR, and the organization of these genes is highly similar (41–43). Both the human and the zebrafish gene encodes two splice variants, the α-isoform, the canonical receptor, and the β-isoform, which has no transcriptional activity (42). The DNA binding domain (DBD) and ligand binding domain (LBD) of the canonical α-isoform of the human and zebrafish GR share similarities of 98.4% and 86.5% respectively (42). The zebrafish GR α-isoform, hereafter referred to as Gr, mediates GC effects that have traditionally been observed in humans and other mammals as well, like the effects on metabolism (44) and the suppression of the immune system (45). This makes the zebrafish an ideal model to study the mechanisms of GC action *in vivo* (46, 47). In a recent study, we have demonstrated that GCs inhibit the activation of the immune system in zebrafish larvae upon wounding (48). Treatment with a synthetic GC attenuated the migration of neutrophils, and inhibited the differentiation of macrophages towards a pro-inflammatory phenotype without affecting the migration of the latter cell type.

In the present study, to investigate the functional consequences of the previously observed GC effects on immune cells, we have investigated how GCs modulate the course of an *Mm* infection in zebrafish larvae. We demonstrate that beclomethasone increases the level of *Mm* infection and tissue dissemination. This increased *Mm* infection can be explained by an inhibition of the phagocytic activity of macrophages by beclomethasone, which did not affect the microbicidal capacity of these cells. The inhibitory effect of beclomethasone on phagocytosis, which most likely results from Gr interfering with the transcription of genes required for phagocytosis, results in a higher percentage of extracellular bacteria, which eventually leads to an exacerbation of the *Mm* infection.

## Materials and Methods

### Zebrafish Lines and Maintenance

Zebrafish were maintained and handled according to the guidelines from the Zebrafish Model Organism Database (zfin.org) and in compliance with the directives of the local animal welfare body of Leiden University. They were exposed to a 14 hours light and 10 hours dark cycle to maintain circadian rhythmicity. Fertilization was performed by natural spawning at the beginning of the light period. Eggs were collected and raised at 28°C in egg water (60 µg/ml Instant Ocean sea salts and 0.0025% methylene blue). The following fish lines were used: wild type strain AB/TL, and the transgenic lines *Tg(mpeg1:mCherry*-*F^umsF001^)* (49) and *Tg(mpeg1:eGFP^gl22^)* (50).

### Bacterial Culture and Infection Through Intravenous Injections

Bacteria used for this study were *Mycobacterium marinum*, strain M, constitutively fluorescently labelled with Wasabi or mCrimson (51, 52), *Mm* mutant *Δerp* labelled with Wasabi (53), *Salmonella enterica serovar* Typhimurium (*S.* Typhimurium) wild type (wt) strain SL1344 labelled with mCherry (54, 55), and a reactive oxygen species (ROS) biosensor *S.* Typhimurium strain (*SL1344 sifBp::mCherry/pkatGp-gfpOVA*) (54, 56). The *Mm* and *S.* Typhimurium strains were cultured at 28°C and 37°C respectively and the bacterial suspensions were prepared with phosphate buffered saline (PBS) with 2% (w/v) polyvinylpyrrolidone-40 (PVP40, Sigma-Aldrich), as previously described (57). The suspension of *Mm Δerp-*Wasabi was prepared directly from -80°C frozen aliquots.

After anesthesia with 0.02% aminobenzoic acid ethyl ester (tricaine, Sigma-Aldrich), 28 hours post fertilization (hpf) embryos were injected with *Mm* or *S.* Typhimurium into the blood island (or hindbrain if specified) under a Leica M165C stereomicroscope, as previously described (57). The injection dose was 200 CFU for *Mm* and 50 CFU for *S.* Typhimurium, except for the experiments to assess activation of the ROS biosensor, where a high bacterial dose of 2000-4000 CFU was used as previously described (56).

### Chemical Treatments and Bacterial Burden Quantification

The embryos were treated with 25 μM (or different if specified) beclomethasone (Sigma-Aldrich) or vehicle (0.05% dimethyl sulfoxide (DMSO)) in egg water from 2 hours before injection to the end of an experiment. RU-486 (Sigma-Aldrich) was administered at a concentration of 5 μM (0.02% DMSO), and cycloheximide (Sigma-Aldrich) at 100 μg/ml (0.04% DMSO). If the treatment lasted longer than 1 day, the medium was refreshed every day.

For bacterial burden quantification, the embryos from the vehicle- and beclomethasone-treated groups were imaged alive using a Leica M205FA fluorescence stereomicroscope equipped with a Leica DFC 345FX camera (Leica Microsystems). The images were analyzed using custom-designed pixel quantification software (previously described by Benard et al. (58), and Image J (plugin ‘Analyze Particles’).

### Hindbrain Infection and Analysis of Dissemination

To assess the dissemination efficiency, the embryos were injected with 50 CFU *Mm* into the hindbrain at 28 hpf. At 2 dpi, the embryos were imaged with a Leica M205FA fluorescence stereomicroscope equipped with a Leica DFC 345FX camera. The embryos were classified into two categories: with or without disseminated infection. An embryo was considered without disseminated infection if all the bacteria were still contained in the hindbrain ventricle and considered with dissemination if bacteria were present in any other part of the embryo.

### Analysis of Microbicidal Activity

After infection at 28 hpf with *Mm Δerp-*Wasabi, *Tg(mpeg1:mCherry-F)* embryos were fixed at 44 hpi with 4% paraformaldehyde (PFA, Sigma-Aldrich) and imaged using a Leica TCS SP8 confocal microscope with 40X objective (NA 1.3). All macrophages that contained *Mm Δerp-*Wasabi in the tail region were analyzed. The level of infection inside macrophages was classified into two categories based on the number of bacteria: 1-10 bacteria or >10 bacteria, following established protocols (59, 60). To quantify the bacterial burden of *Mm Δerp* infected embryos, the fluorescence intensity of the Wasabi signal was measured using image J.

### Analysis of Phagocytic Activity

To study the dynamics of bacterial phagocytosis during the early stage of infection, *Tg(mpeg1:mCherry-F)* or *Tg(mpeg1:eGFP)* embryos were intravenously infected at 28 hpf with *Mm*-Wasabi or *S.* Typhimurium-mCherry. At 5, 15 and 25 min after infection the embryos were fixed with 4% PFA, so they could later be imaged using a Leica TCS SP8 confocal microscope with 20X objective (NA 0.75). The yolk sac area was selected as the quantification area (**Figure 3A**). The number of fluorescently labelled *Mm* or *S.* Typhimurium in this area, and those present inside a macrophage, were counted in a manual and blinded way.

### TUNEL Assay

After infection at 28 hpf, *Tg(mpeg1:mCherry-F)* embryos were fixed with 4% PFA at 48 hpi and stained using terminal deoxynucleotidyl transferase dUTP nick end labelling (TUNEL) with the In Situ Cell Death Detection Kit, TMR red (Sigma-Aldrich), as previously described by Zhang et al. (2019). For this TUNEL staining, the embryos were first dehydrated and then rehydrated gradually with methanol in PBS, and permeabilized with 10 μg/ml Proteinase K (Roche). The embryos were subsequently fixed with 4% PFA for another 20 min and stained with reagent mixture overnight at 37°C. After the reaction was stopped by washing with PBS containing 0.05% Tween-20 (PBST), the caudal hematopoietic tissue (CHT) region of the embryos was imaged using a Leica TCS SP8 confocal microscope with 40X objective (NA 1.3). The total number of fluorescently labelled *Mm* clusters and the number of these clusters overlapping with TUNEL staining were counted in a manual and blinded way.

### Fluorescence-Activated Cell Sorting (FACS) of Macrophages

Macrophages were sorted from *Tg(mpeg1:mCherry-F)* embryos as previously described (61, 62). Dissociation was performed with 150-200 embryos for each sample after 2 hours beclomethasone or vehicle treatment (started at 28 hpf) using Liberase TL (Roche) and stopped by adding Fetal Calf Serum (FCS) to a final concentration of 10%. Isolated cells were resuspended in Dulbecco’s PBS (DPBS), and filtered through a 40 μm cell strainer. Actinomycin D (Sigma-Aldrich) was added (final concentration of 1 µg/ml) to each step to inhibit transcription. Macrophages were sorted based on their red fluorescent signal using a FACSAria III cell sorter (BD Biosciences). The sorted cells were collected in QIAzol lysis reagent (Qiagen) for RNA isolation.

### RNA Isolation, cDNA synthesis and Quantitative PCR (qPCR) Analysis

RNA isolation from FACS-sorted cells was performed using the miRNeasy mini kit (Qiagen), according to the manufacturer’s instructions. Extracted total RNA was reverse-transcribed using the iScript™ cDNA Synthesis Kit (Bio-Rad). QPCR was performed on a MyiQ Single-Color Real-Time PCR Detection System (Bio-Rad) using iTaq™ Universal SYBR^®^ Green Supermix (Bio-Rad). The sequences of the primers used are provided in **Supplementary Table 1**. Cycling conditions were pre-denaturation for 3 min at 95°C, followed by 40 cycles of denaturation for 15 s at 95°C, annealing for 30 s at 60°C, and elongation for 30 s at 72°C. Fluorescent signals were measured at the end of each cycle. Cycle threshold values (Ct values, i.e. the cycle numbers at which a threshold value of the fluorescence intensity was reached) were determined for each sample. To determine the gene regulation due to beclomethasone treatment in each experiment, the average Ct value of the beclomethasone treated samples was subtracted from the average Ct value of the vehicle-treated samples, and the fold change of gene expression was calculated, which was subsequently adjusted to the expression levels of a reference gene (*peptidylprolyl isomerase Ab* (*ppiab*)).

### Analysis of ROS Production

Embryos were infected at 28 hpf with a ROS biosensor *S.* Typhimurium strain (*SL1344 sifBp::mCherry/pkatGp-gfpOVA*) which constitutively expresses mCherry, while the expression of an unstable GFP variant (GFP-OVA) is under the control of an OxyR-activated promoter, which is activated upon exposure to ROS from the host (54). Embryos were imaged using a Leica TCS SP8 confocal microscope with 40X objective (NA 1.3) at 4 hpi. An area in the CHT region, close to the site of injection, was selected for analysis. The fluorescence intensity of the GFP signal in this area, which represents ROS production, and the intensity of the mCherry signal from *S.* Typhimurium were quantified, and their ratio was calculated.

### Statistical Analysis

**Figure 1:**
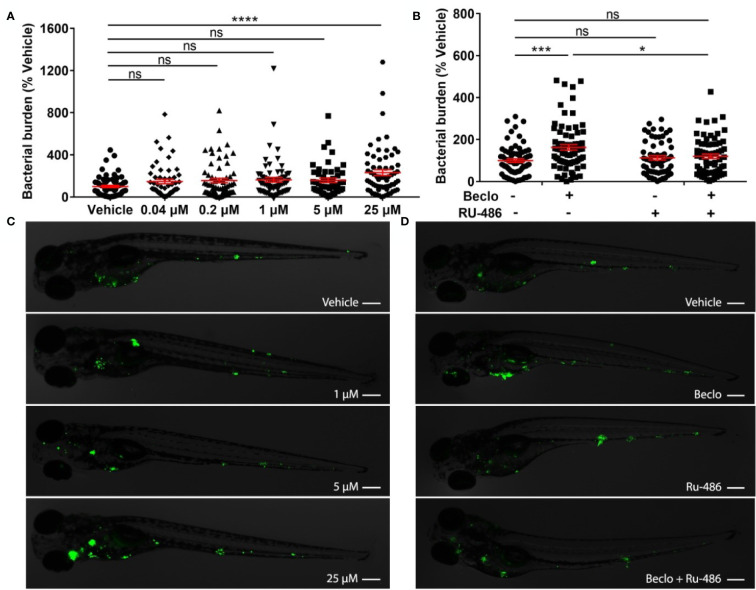
Effect of beclomethasone on *Mm* infection burden in zebrafish. **(A)** Bacterial burden of zebrafish larvae at four days after intravenous injection (at 28 hpf) of *Mm* and treatment with vehicle or different concentrations of beclomethasone (beclo), started at 2 h before infection. Statistical analysis by one-way ANOVA with Bonferroni’s *post hoc* test revealed that the bacterial burden was significantly increased in the group treated with 25 µM beclomethasone, compared to the burden of the vehicle-treated group. **(B)** Effect of the GR antagonist RU-486 on the beclomethasone-induced increase of the bacterial burden at 4 dpi. The bacterial burden was significantly increased by beclomethasone (25 µM) treatment and this increase was abolished in the presence of RU-486. Statistical analysis was performed by two-way ANOVA with Tukey’s *post hoc* test. In panels **(A, B)**, each data point represents a single larva and the means ± s.e.m. of data accumulated from three independent experiments are shown in red. Statistical significance is indicated by: ns, non-significant; *P<0.05; ***P<0.001; ****P<0.0001. **(C, D)** Representative fluorescence microscopy images of *Mm*-infected larvae at 4 days post infection (dpi), representing experimental groups presented in panels **(A, B)** Bacteria are shown in green. Scale bar = 200 μm.

**Figure 2:**
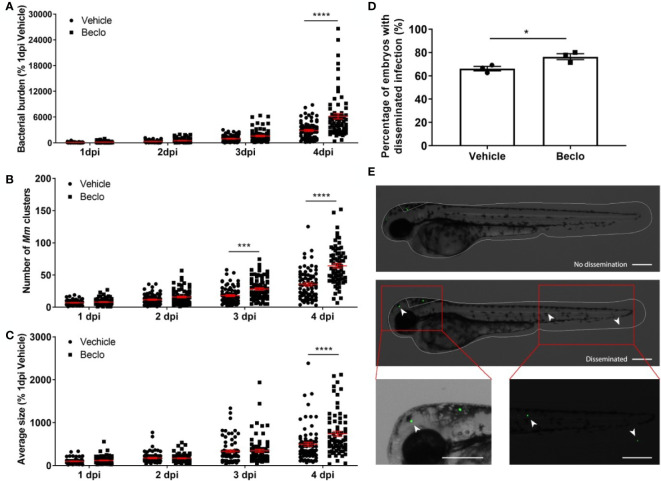
Beclomethasone effects on *Mm* infection progression and bacterial dissemination. **(A–C)**. Bacterial burden **(A)**, number of bacterial clusters **(B)** and the average size of bacterial clusters **(C)** were determined at 1, 2, 3 and 4 dpi following intravenous *Mm* injection (28 hpf) and treatment with vehicle or 25 µM beclomethasone, started at 2 h before infection. Significant increases due to the beclomethasone treatment were observed for all parameters at 4 dpi. For the number of bacterial clusters, the increase was also significant at 3 dpi. Statistical analysis was performed by two-way ANOVA with Tukey’s *post hoc* test. Each data point represents a single larva and the means ± s.e.m. of data accumulated from three independent experiments are shown in red. Statistical significance is indicated by: ***P<0.001; ****P<0.0001. **(D)** Effect of beclomethasone on dissemination of *Mm* by hindbrain ventricle injection. Hindbrain infections were performed at 28 hpf, and at 24 hours post infection (hpi), a significantly increased percentage of larvae with disseminated Mm infection was detected in the beclomethasone-treated group compared to the vehicle group. Statistical analysis was performed by two-tailed t-test. Values shown are the means ± s.e.m. of three independent experiments with a total sample size of 27 in the vehicle-treated group and 31 in the beclomethasone-treated group. Statistical significance is indicated by: *P<0.05. **(E)** Representative images of embryos with and without dissemination of the infection upon hindbrain injection of *Mm*. Scale bar = 200 μm.

**Figure 3:**
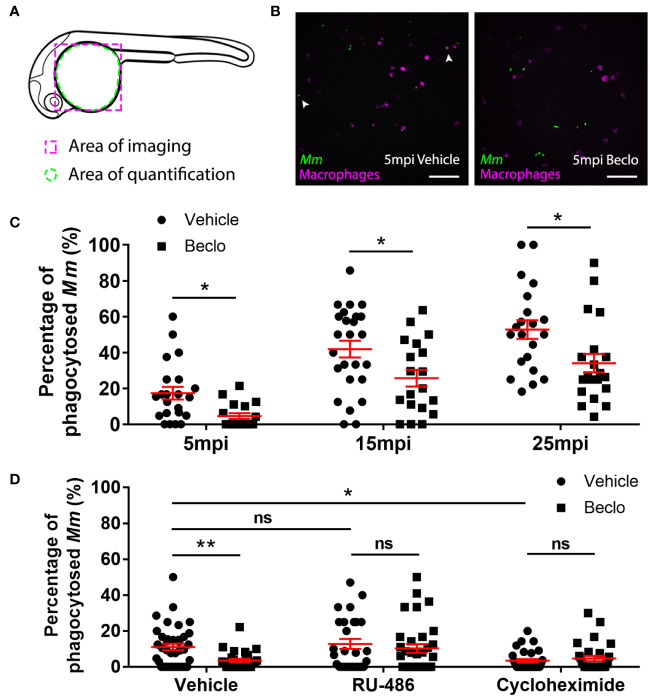
Effect of beclomethasone on phagocytic activity of macrophages and its dependency on Gr and *de novo* protein synthesis. **(A)** Schematic drawing of a zebrafish embryos at 28 hpf indicating the areas of imaging (purple dashed box, used for representative images) and quantification (green dashed circle) of *Mm* phagocytosis. **(B)** Representative confocal microscopy images of embryos of the *Tg(mpeg1:mCherry-F)* line injected with Mm at 28 hpf. Images were taken of infected embryos that were vehicle- or beclomethasone-treated at 5 minutes post infection (mpi). Macrophages are shown in magenta, bacteria in green. Scale bar = 100 μm. Arrowheads indicate bacterial clusters phagocytosed by macrophages. **(C)** Percentages of phagocytosed *Mm* clusters (of total number of *Mm* clusters) at 5, 15 and 25 mpi. Statistical analysis, performed by fitting data to a beta inflated regression with Tukey’s *post hoc* test, showed that beclomethasone decreased this percentage at all three time points. **(D)** Effects of RU-486 and cycloheximide on the beclomethasone-inhibited phagocytic activity. Embryos were treated with vehicle or beclomethasone and received either a vehicle, RU-486 or cycloheximide co-treatment two hours before injection of *Mm* at 28 hpf, and phagocytic activity was determined at 5 mpi. The significant inhibitory effect of beclomethasone on phagocytosis was not observed in the presence of RU-486. Cycloheximide, just like beclomethasone, significantly inhibited the phagocytic activity, and the combined cycloheximide/beclomethasone treatment showed the same level of inhibition. Statistical analysis was performed by fitting data to a beta inflated regression with Tukey’s *post hoc* test. In panels **(C, D)**, each data point represents a single embryo and the means ± s.e.m. of data accumulated from three independent experiments are shown in red. Statistical significance is indicated by: ns, non-significant; *P<0.05; **P<0.01.

**Figure 4:**
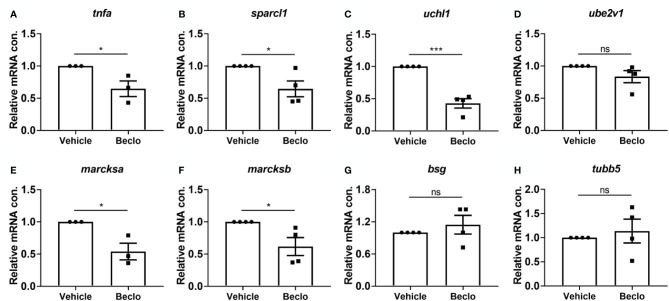
Effect of beclomethasone on gene expression levels in FACS-sorted macrophages. At 28 hpf, *Tg(mpeg1:mCherry-F)* embryos were treated with vehicle or beclomethasone for two hours, after which macrophages were isolated by FACS sorting. Gene expression levels were determined in the sorted cells by qPCR for *tnfa* **(A)**, *sparcl1* **(B)**, *uchl1* **(C)**, *ube2v1* **(D)**, *marcksa* **(E)**, *marcksb* **(F)**, *bsg* **(G)** and *tubb5* **(H)**. Statistical analysis by two-tailed t-test showed that the levels of *tnfa*, *sparcl1*, *uchl1*, *marcksa* and *marcksb* expression were significantly inhibited by beclomethasone treatment. Data shown are the means ± s.e.m. of three or four independent experiments, and markers show averages of individual experiments. Statistical significance is indicated by: ns, non-significant; *P<0.05; ***P<0.001.

**Figure 5:**
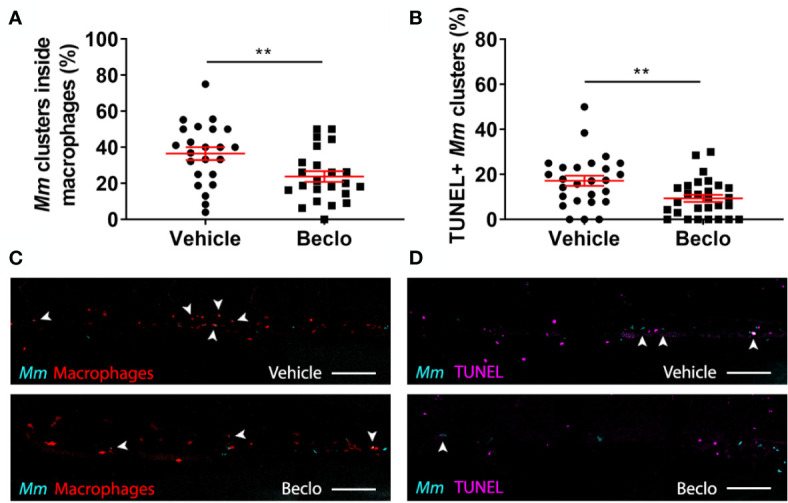
Effect of beclomethasone on intracellular bacterial growth and cell death. Infection was performed in *Tg(mpeg1:eGFP)* embryos at 28 hpf, a TUNEL assay was performed at 48 hpi, and the CHT region of the embryos was imaged using confocal microscopy. **(A)** The percentage of *Mm* clusters that were inside macrophages based on colocalization with the green fluorescent signal from eGFP. Statistical analysis was performed by two-tailed t-test. In the beclomethasone-treated group, the percentage of *Mm* clusters inside macrophages was significantly lower compared to the vehicle-treated group. **(B)** The percentage of TUNEL-positive *Mm* clusters. Statistical analysis by two-tailed t-test showed that the beclomethasone-treated group had a significantly lower percentage of TUNEL+ *Mm* clusters. In panels **(A, B)**, each data point represents a single embryo and the means ± s.e.m. of data accumulated from three independent experiments are shown in red. Statistical significance is indicated by: **P<0.01. **(C)** Representative confocal microscopy images of macrophage phagocytosis. Bacteria are shown in blue and macrophages in red. Arrowheads indicate intracellular bacterial clusters. Scale bar = 100 μm. **(D)** Representative confocal microscopy images of cell death (TUNEL+ cells in magenta) and *Mm* infection (bacteria in blue). Arrowheads indicate bacterial clusters overlapping with TUNEL+ cells. Scale bar = 100 μm.

**Figure 6:**
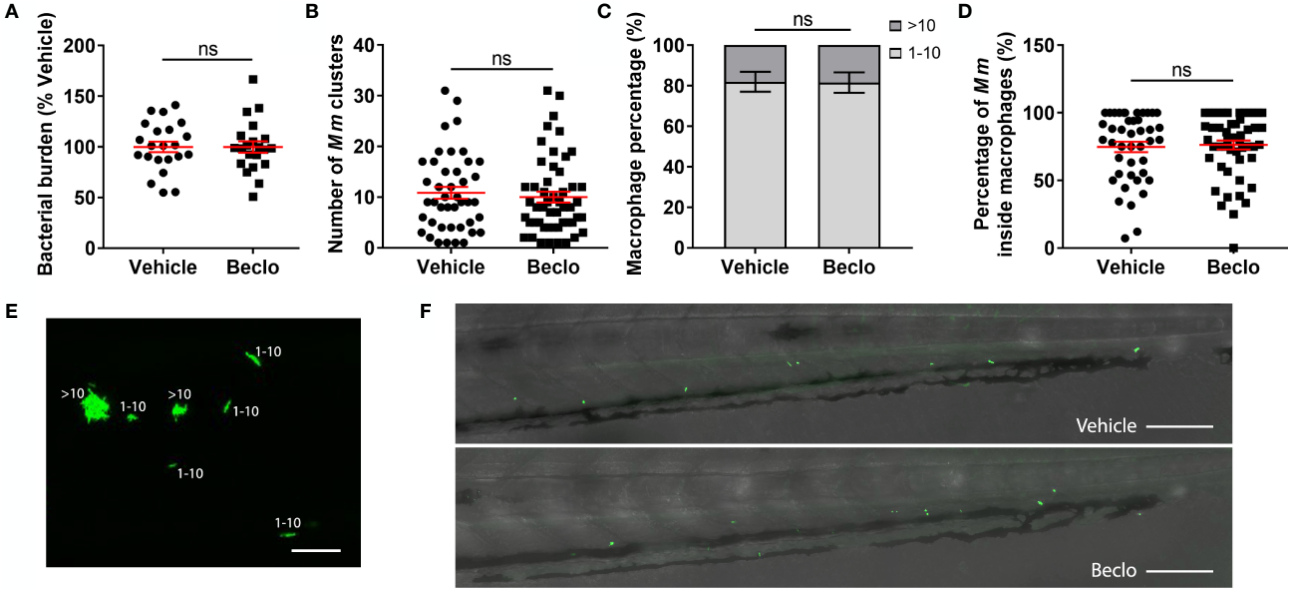
Effect of beclomethasone on the bacterial growth of the *Mm Δerp* mutant. The *Mm Δerp* mutant strain was injected intravenously at 28 hpf, and at 44 hpi, the bacterial burden **(A)**, the number of *Mm* clusters **(B)**, the percentage of macrophages that contained 1-10 or more than 10 bacteria (of all macrophages containing bacteria) **(C)**, and the percentage of *Mm* inside macrophages **(D)** were determined. No significant difference was observed between the vehicle- and beclomethasone-treated groups for any of these parameters. Statistical analysis was performed using two-tailed t-tests. Values shown are the means ± s.e.m. of three independent experiments, with each data point representing a single embryo. Statistical significance is indicated by: ns, non-significant. **(E)** Representative confocal microscopy image of *Mm Δerp* bacterial clusters (bacteria in green), indicated are clusters containing 1-10 bacteria and clusters containing more than 10 bacteria. Scale bar = 20 μm. **(F)** Representative images of the tail regions of a vehicle- and a beclomethasone-treated embryo infected with *Mm Δerp* bacteria. Scale bar = 100 μm.

**Figure 7:**
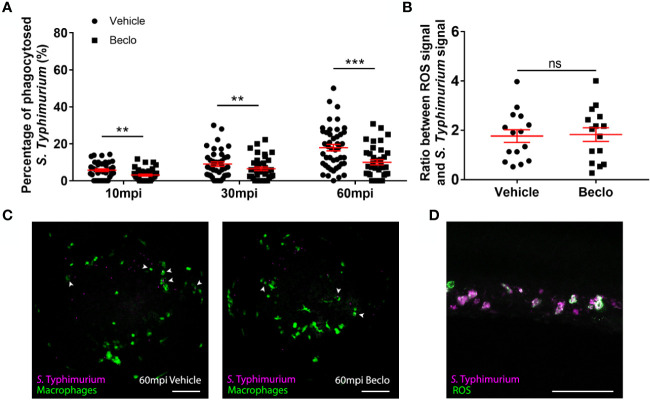
Effect of beclomethasone on phagocytosis of *Salmonella* Typhimurium and ROS production. At 28 hpf *Tg(mpeg1:eGFP)* embryos (vehicle- or beclomethasone-treated) were infected with *S.* Typhimurium through intravenous injection. **(A)** Percentage of phagocytosed *S.* Typhimurium wt at 10, 30 and 60 mpi. Confocal microscopy images were taken of the yolk area (as indicated in **Figure 3A**), and the percentage of bacteria inside macrophages was determined. Statistical analysis, performed by fitting data to a beta-inflated regression with Tukey’s *post hoc* test, showed that the phagocytic activity of macrophages was significantly inhibited by beclomethasone treatment at 60 mpi, and not at other time points. **(B)** Ratio between ROS signal and *S.* Typhimurium signal. Embryos were injected with a ROS biosensor strain. At 4 hpi, confocal microscopy images were taken of the CHT region and the ratio between the ROS-induced fluorescent signal and the signal from a constitutively expressed fluorescent protein in the bacteria was determined. Statistical analysis was performed by two-tailed t-test, indicating no significant difference between the two vehicle- and beclomethasone-treated groups. In panel **(A, B)**, each data point represents a single embryo and the means ± s.e.m. of data accumulated from three independent experiments are shown in red. Statistical significance is indicated by: ns, non-significant; **P<0.01; ***P<0.001. **(C)** Representative confocal microscopy images of *S.* Typhimurium wt infected vehicle- and beclomethasone-treated individuals at 60 mpi. Bacteria are shown in magenta, macrophages in green. Arrowheads indicate bacteria phagocytosed by macrophages. Scale bar = 100 μm. **(D)** Representative confocal microscopy image of a vehicle-treated embryo infected with the *S.* Typhimurium ROS biosensor strain, showing the bacterial signal (magenta) and the ROS biosensor signal (green). Scale bar = 100 μm.

Statistical analysis was performed using GraphPad Prism by one-way ANOVA with Bonferroni’s *post hoc* test (**Figure 1A**) or two-way ANOVA with Tukey’s *post hoc* test (**Figures 1B** and **2A–C**) or two-tailed t-test (**Figures 2D**, **4**–**6** and **7B**) or using R Statistical Software by fitting data to a beta inflated regression (from ‘gamlss’ package) (63) with Tukey’s *post hoc* test (**Figures 3** and **7A**).

## Results

### Beclomethasone Increases Mycobacterial Infection Through Glucocorticoid Receptor (Gr) Activation

To study the effect of GC treatment on *Mm* infection in zebrafish, we pretreated zebrafish embryos with beclomethasone and infected them intravenously with fluorescently labelled *Mm*. At 4 days post infection (dpi), the bacterial burden was assessed by quantification of pixel intensities of fluorescence microscopy images. We found that the bacterial burden increased by 2.3 fold when embryos were treated with 25 μM beclomethasone compared with the vehicle-treated group (**Figures 1A, C**). Beclomethasone treatment at lower concentrations of 0.04, 0.2, 1 and 5 μM did not affect the bacterial burden. Therefore, a concentration of 25 μM beclomethasone was used in subsequent experiments. We have previously shown that this concentration effectively reduces wound-induced neutrophil migration in zebrafish as well (48).

To demonstrate that the beclomethasone-induced increase in bacterial burden was not due to a general toxicity of beclomethasone but mediated specifically by the Gr, we used the GR antagonist RU-486. The results of these experiments showed that the beclomethasone-induced increase in bacterial burden at 4 dpi was abolished when co-treatment with RU-486 was applied (**Figures 1B, D**), which indicates that the effect of beclomethasone requires activation of Gr. No significant difference was observed when the RU-486-treated larvae were compared to the vehicle-treated group. In conclusion, beclomethasone increases the level of *Mm* infection in zebrafish larvae and this effect is mediated by Gr.

### Beclomethasone Treatment Leads to a Higher Infection and Dissemination Level

Subsequently, we analyzed the effect of beclomethasone on *Mm* infection in more detail. The total bacterial burden (**Figure 2A**), the number of bacterial clusters per individual (**Figure 2B**) and the average size of the bacterial clusters (**Figure 2C**) were quantified at 1, 2, 3 and 4 dpi. The results showed that the difference in bacterial burden between the beclomethasone-treated group and the vehicle group was not significant at 1-3 dpi, but that a significant difference was observed at 4 dpi (6186.1 ± 626.5 *vs* 2870.5 ± 235.0). However, a significant increase in the number of bacterial clusters in the beclomethasone-treated group was already detected at 3 dpi (28.3 ± 1.9 vs 18.1 ± 1.5 in the vehicle group) which was sustained at 4 dpi (64.2 ± 3.5 *vs* 35.4 ± 2.6). The size of the bacterial clusters at 4 dpi was also increased in the beclomethasone-treated group compared to the cluster size in the vehicle-treated group (741.6 ± 58.3 *vs* 498.3 ± 45.7). The increase in the number of bacterial clusters indicates an increased dissemination of the infection due to beclomethasone treatment. We confirmed this effect of beclomethasone on bacterial dissemination using hindbrain infection (**Figures 2D, E**). Following *Mm* injection into the hindbrain ventricle, 66.1 ± 2.0% of embryos in the vehicle-treated group showed disseminated infection in tissues of the head and tail at 24 hours post infection (hpi), while a significantly higher number (76.4 ± 2.6%) showed this dissemination in the beclomethasone-treated group.

### Beclomethasone Activation of Gr Inhibits Macrophage Phagocytic Activity

Since previous studies showed that increased *Mm* infection could be related to decreased phagocytic activity of macrophages in zebrafish (64), we studied the effect of beclomethasone on phagocytosis. We used the *Tg(mpeg1:mCherry-F)* line in which macrophages are fluorescently labeled, and assessed phagocytic activity of macrophages by determining the percentage of *Mm* that were internalized by macrophages in the yolk sac area within 5-25 min after infection based on previous analysis of the kinetics of this response (64) (**Figures 3A–C**). The bacteria are intravenously injected at 28 hpf, and at this developmental stage the primitive macrophages of the zebrafish embryos are primarily localized in the blood circulation (65). Phagocytosis of the injected bacteria is therefore independent of tissue migration of macrophages in this experiment. In the vehicle-treated group, the percentage of phagocytosed *Mm* was 17.4 ± 3.5% at 5 minutes post infection (mpi) and gradually increased to 41.9 ± 4.9% and 52.8 ± 5.2% at 15 and 25 mpi respectively. At each of these time points, a lower percentage of *Mm* were phagocytosed in the beclomethasone-treated group (4.6 ± 1.6% at 5 mpi, 25.7 ± 4.7% at 15 mpi and 34.0 ± 5.2% at 25 mpi). In addition, we studied the involvement of Gr in the beclomethasone-induced inhibition of phagocytosis at 5 mpi, by co-treatment with the GR antagonist RU-486 (**Figure 3D**). We found that the decreased phagocytic activity that was observed upon beclomethasone treatment was abolished when larvae were co-treated with RU-486, indicating that the inhibition of phagocytosis by beclomethasone is mediated by Gr.

Gr generally acts as a transcription factor, modulating the transcription rate of a wide variety of genes. To study whether phagocytosis could be modulated by altering the process of protein synthesis, we blocked *de novo* protein synthesis by treatment with cycloheximide (**Figure 3D**). We observed that the phagocytic activity of macrophages at 5 mpi was decreased by the cycloheximide treatment (3.4 ± 1.0% vs 11.1 ± 1.8% in the vehicle group). These data demonstrate that phagocytosis depends on *de novo* protein synthesis, and indicate that repression of transcription of specific genes may inhibit the phagocytic activity of macrophages.

### Beclomethasone Inhibits Phagocytosis-Related Gene Expression in Macrophages

To unravel the molecular mechanisms underlying the beclomethasone-induced inhibition of the phagocytic activity of macrophages, we performed qPCR analysis on FACS-sorted macrophages derived from 28 hpf larvae after 2 h of beclomethasone treatment. To determine the phenotype of the sorted macrophages, the expression of a classic pro-inflammatory gene, *tnfa*, was measured (66, 67). The levels of *tnfa* expression were significantly lower after beclomethasone treatment (**Figure 4A**), in agreement with previously reported transcriptome analysis (48). In addition, we measured the expression levels of seven genes, for which a phagocytosis-promoting role has been established: *sparcl1*, *uchl1*, *ube2v1*, *marcksa*, *marcksb*, *bsg* and *tubb5* (68–70) (**Figures 4B–H**). The expression levels of four of these genes, *sparcl1*, *uchl1*, *marcksa* and *marcksb*, were inhibited by beclomethasone treatment, while the levels of the other three (*ube2v1, bsg* and *tubb5)* were not affected. These data suggest that beclomethasone inhibits the phagocytic activity of macrophages by suppressing the transcription of phagocytosis-related genes in these cells.

### Beclomethasone Treatment Results in Fewer Intracellular Bacteria, Limits Infected Cell Death, Without Affecting the Microbicidal Capacity of Macrophages

To further analyze the possible mechanisms underlying the beclomethasone-induced increase in the *Mm* infection level, we assessed the percentage of bacteria that are present inside and outside macrophages in the caudal hematopoietic tissue (CHT) at 48 hpi using *Mm* infection in the *Tg(mpeg1:eGFP)* line. The results showed that beclomethasone treatment resulted in a decreased percentage of intracellular bacteria (23.8 ± 3.0%) compared to the percentage in the vehicle-treated group (36.5 ± 3.6%) (**Figures 5A, C**). This result was in line with the observed decrease in phagocytosis at earlier stages of infection. To determine if the lower percentage of intracellular bacteria correlated with higher macrophage viability, we used terminal deoxynucleotidyl transferase dUTP nick end labelling (TUNEL) staining to detect cell death (71). In the beclomethasone-treated group, the percentage of *Mm* that were colocalized with TUNEL staining (9.4 ± 1.6%) at 48 hpi was significantly lower compared to the percentage of the vehicle group (17.2 ± 2.3%) (**Figures 5B, D**). These data suggest that the observed inhibition of phagocytosis upon beclomethasone treatment causes a decrease in the percentage of intracellular bacteria, which underlies the lower numbers of macrophages undergoing cell death as a result of the *Mm* infection.

Next, we considered the possibility that beclomethasone, in addition to inhibiting phagocytosis, might affect the microbicidal capacity of macrophages. Therefore, we injected embryos with bacteria from the attenuated *Mm Δerp* strain. The *erp* (exported repetitive protein) virulence factor is required for bacteria to survive and replicate inside acidic compartments. These *Δerp* bacteria are therefore deficient for growth inside macrophages, and assessing their numbers is considered a readout for the clearance capacity of the host, since the initial infection dose is not increased in the absence of bacterial replication (59, 60). No significant difference was observed at 44 hpi for the bacterial burden, the number of *Mm* clusters, the percentage of bacteria-containing macrophages that contained only 1-10 bacteria and the percentage of *Mm* inside macrophage in the tail region between the beclomethasone-treated group and the vehicle-treated group (**Figure 6**). These findings indicate that beclomethasone treatment does not lead to an altered microbicidal capacity of macrophages. Taken together, our results identify the inhibition of phagocytosis as the primary effect of beclomethasone during mycobacterial infection of zebrafish, which would exacerbate infection with *Mm* wt due to increased extracellular bacterial growth in line with previous results (72).

### Beclomethasone Inhibits Macrophage Phagocytosis of *Salmonella* Typhimurium and Does Not Affect Reactive Oxygen Species (ROS) Production

To study whether the beclomethasone-induced inhibitory effect on macrophage phagocytosis of *Mm* can be generalized to other bacterial infections, we analyzed the effect of beclomethasone on infection with *Salmonella* Typhimurium (*S.* Typhimurium), which is also an intracellular pathogen, but belongs to the gram-negative class. We quantified the percentages of bacteria phagocytosed by macrophages at different time points after infection in the *Tg(mpeg1:eGFP)* fish line (**Figures 7A, C**). In the vehicle group, the percentage of phagocytosed *S.* Typhimurium increased from 5.7 ± 0.7% at 10 mpi to 9.0 ± 1.2% at 30 mpi and 17.9 ± 1.7% at 60 mpi, and these percentages were significantly lower in the beclomethasone-treated group at all time points (3.1 ± 0.5% at 10 mpi, 6.5 ± 1.0% at 30 mpi and 10.0 ± 1.4% at 60 mpi). These data demonstrate that the inhibitory effect of beclomethasone on the phagocytic activity of macrophages is not specific for *Mm*, but can also be observed for a distantly related *Salmonella* species.

In order to further investigate the effect of GC treatment on the microbicidal activity of host immune cells, we analyzed the effect of beclomethasone on reactive oxygen species (ROS) production upon *S.* Typhimurium infection. To analyze ROS production, we used a ROS biosensor *S.* Typhimurium strain, which expresses GFP when it encounters ROS (54, 56). At 4 hpi, the ROS-activated fluorescent signals from the bacteria were determined and the ratio between this signal and a signal from mCherry that was constitutively expressed by the bacteria, was calculated for both vehicle- and beclomethasone-treated embryos (**Figures 7B, D**). No significant difference was observed between the two groups, indicating no effect of beclomethasone on the ROS production in immune cells upon a bacterial infection.

In conclusion, beclomethasone inhibits the phagocytosis of both Mycobacteria and Salmonella by macrophages in the zebrafish host, while no evidence for additional effects on microbicidal responses was found in either case.

## Discussion

Synthetic GCs are widely prescribed to treat various immune-related diseases, but their clinical use is limited by the severe side effects evoked by prolonged therapy, including a higher susceptibility to TB (5, 14). In order to gain more insight into the mechanism underlying this GC effect, we used the zebrafish *Mm* infection model, which mimics human TB, and studied the effect of GC treatment on the development of the infection. We showed that GC treatment increased the level of *Mm* infection, which was reflected in the overall bacterial burden, the size and number of bacterial clusters and the level of dissemination. Since we found that GC treatment inhibited the phagocytic activity but not the microbicidal capacity of macrophages, we suggest that the GC-induced increase in infection susceptibility is due to the inhibition on phagocytosis. Analysis of the transcription level of phagocytosis-related genes in macrophages suggested that the inhibition of phagocytic activity by GCs is mediated by Gr interfering with phagocytosis-related gene transcription. The lower phagocytic activity of the macrophages probably underlies the decrease in the percentage of intracellular bacteria that we observed, probably resulting in a lower level of cell death due to the *Mm* infection and exacerbated growth of the extracellular bacterial fraction. Finally, we showed that GC treatment not only limited phagocytosis of mycobacteria, but also of a Salmonella species, which suggests that the decrease in phagocytic activity may also explain the increased susceptibility to other bacterial infections that is commonly observed in patients receiving GC therapy (3–5).

Upon bacterial infections, macrophages are the first responders of the immune system. In humans, *Mtb* generally infects lungs due to its air transmission properties and in the lungs it is taken up by alveolar macrophages within the first few days. In later stages, *Mtb* replicates, translocates to secondary loci and aggregates into granulomas with other attracted immune cells (73–75). Consistently, in the zebrafish model, *Mm* is predominantly phagocytosed by macrophages within 30-60 min after intravenous infection in embryos, leading to initial stages of granuloma formation in the next few days (37, 64). The phagocytosis activity and microbicidal capacity of macrophages have been shown to be important for dealing with *Mm* infection (64, 72). Interestingly, in our study we found that the microbicidal capacity of macrophages (determined using the *Mm Δerp* and the *St* biosensor strains) was not affected by GC treatment, which suggests that the inhibition of macrophage phagocytosis is a specific effect of GCs targeted at the uptake of pathogens rather than a global suppression of anti-microbial processes in macrophages.

Our study in the zebrafish model provides *in vivo* evidence for GC interference with macrophage phagocytosis. In line with our results, it has previously been shown that GCs decrease the phagocytosis of several *Escherichia coli* strains by human monocyte-derived (THP-1) macrophages and by murine bone marrow-derived macrophages (BMDMs) (76). Similarly to our results, in this study the reduced phagocytosis activity was accompanied by a decreased expression of genes involved in phagosome formation including *MARCKS* and pro-inflammatory genes like *TNF* (76). In earlier studies, decreased macrophage phagocytosis of carbon particles was observed *in vivo*, in GC-treated rats and rheumatoid arthritis patients (77, 78). However, in other studies GC treatment has been shown to enhance the bacterial phagocytosis by macrophages. Upon GC exposure, increased bacterial phagocytic activity of human monocyte-derived macrophages was observed for *Haemophillus influenzae* and *Streptococcus pneumoniae* (79), and *Staphylococcus aureus* (80).

It could be argued that the observed effect of beclomethasone on phagocytosis, which looks more like a delay in this process than an inhibition, is unlikely to be solely responsible for the increased bacterial burden that we observed. It must be noted however that for the *Mm* infection, this burden is a result of a continuous cycle of phagocytosis, bacterial replication, cell death and subsequent phagocytosis [i.e. efferocytosis (81)], in which an effect of beclomethasone on phagocytosis may well accumulate over time. For the infection with the avirulent *Δerp* bacteria, which are simply phagocytosed and subsequently cleared, such an accumulation of the beclomethasone effect would be absent. In line with this notion, the percentage of *Mm Δerp* inside macrophages was not different between the vehicle- and the beclomethasone-treated group in our study.

Alternatively, beclomethasone may alter additional processes during the course of the infection contributing to the increased bacterial burden. One such process that can be affected by GC treatment is the migration of macrophages towards a site of infection, and such an effect would in many experimental designs be difficult to separate from effects on phagocytosis. However, in our experiments we have infected the embryos at 28 hpf by intravenous injection of bacteria, upon which all bacteria are rapidly phagocytozed by monocytes/macrophages in the bloodstream (37, 64). Migration does not play a role in this process, because the primitive macrophages of the embryo are almost all located in the circulation at this stage (65). In addition, we have previously shown that beclomethasone does not affect migration of macrophages in a wounding-induced inflammation model (48), which makes an effect of GCs on migration unlikely, although we cannot exclude a possible effect of GCs on macrophage migration in the context of an infection.

Macrophages are often divided into two functional phenotypes: a classically activated phenotype (often referred to as M1) which contributes to the anti-microbial and inflammatory response, and an alternatively activated phenotype (often referred to as M2), which can be subdivided in several different phenotypes which have been shown to be involved in the resolution of inflammation and in wound healing (67, 82). It has been well established that GCs inhibit the differentiation towards the classically activated M1 phenotype (83–86), and we have recently demonstrated in zebrafish larvae that GC treatment inhibits the differentiation of macrophages towards this pro-inflammatory phenotype upon wounding of the tail fin (48). Increased expression of *tnfa* and phagocytic activity are commonly recognized as characteristics of M1-differentiated macrophages, so the GC-induced decreases in *tnfa* expression and phagocytosis that we have observed in the present study are well in line with the inhibitory effect of GCs on the differentiation of macrophages towards an M1 phenotype.

In addition, GCs have been demonstrated to enhance the differentiation of macrophages towards a specific, alternatively activated, anti-inflammatory phenotype, which is considered to play an important role in GCs actively promoting the resolution of inflammation (84, 87). Interestingly, this phenotype shows an enhancement of phagocytic activity, but in the GC-induced differentiation status this activity is nonphlogistic and directed at apoptotic leukocytes, thereby contributing to the resolution of the inflammation. The GC-enhanced phagocytosis of apoptotic neutrophils has been observed in differentiated THP-1 macrophages, through stimulation of a protein S/Mer tyrosine kinase dependent pathway (88–90), and in mouse alveolar macrophages (91).

Our study revealed an inhibitory effect of GCs on four phagocytosis-related genes in FACS-sorted macrophages: *sparcl-1*, *uchl-1*, *marcksa and marcksb*. Among those genes, the human and mouse homologs of *sparcl-1* and *uchl-1* were reported to have a phagocytosis-promoting activity (68, 70). In human THP-1-derived macrophages, MARCKS plays a role in cytoskeletal remodeling and phagosome formation (69). In these cells, the *MARCKS* gene expression was found to be inhibited by dexamethasone treatment, which indicates that GCs induce this effect by activating GRs in macrophages (76). According to the Transcription Factor Targets Dataset generated by Encyclopedia of DNA Elements (ENCODE) project (92), the human genes *SPARCL1*, *UCHL1*, *UCHL5* and *MARCKS* contain binding sites for GR, as determined by Chromatin Immunoprecipitation (ChIP) sequencing analysis. Together with our observation that phagocytosis is dependent on *de novo* protein synthesis, these results support the idea that GC treatment inhibits the phagocytosis activity of macrophages through GRs directly interfering with transcription of genes that stimulate the phagocytic activity of these cells.

After internalization by macrophages, *Mm* are exposed to a bactericidal environment (93). Some bacteria may be killed by macrophages, while others may proliferate mediated by virulence determinants like Erp and RD1 (59, 93, 94). When the macrophages are incapable of containing the bacteria, they undergo cell death leading to recruitment of more macrophages (38). In our study, GC treatment led to a lower percentage of intracellular *Mm* at later stages, consistent with the decreased phagocytosis at early time points, and less *Mm*-related cell death, probably as a result of the decreased number on intracellular bacteria. The GC treatment may also directly affect cell death, since in a recent study it was demonstrated that GCs inhibit necrosis of various *Mtb* infected mouse and human cell types by activating MKP-1, which suppresses a pathway involving p38 MAPK activation ultimately leading to a loss of mitochondrial integrity (95). The increased numbers of extracellular bacteria could traverse endothelial barriers directly and grow more rapidly in a less restrictive environment outside macrophages, which may explain our observation of a higher bacterial burden induced by GC treatment.

Based on our results, it may seem surprising that adjunctive GC therapy is often beneficial to TB patients, and even increases survival among tuberculous meningitis and pericarditis patients (24–26). However, many of these observed beneficial effects are either minor or under debate. This may be due to GC therapy benefiting only a subset of patients whose disease has mainly progressed as a result of an excessive inflammatory response (which can be controlled with GC therapy), rather than a failed reaction to the infection, which was demonstrated for GC-treated TB meningitis patients with specific polymorphisms in the *LTA4H* gene (32). We therefore suggest that in a subset of patients at later stages of infection, the anti-inflammatory effects of a GC treatment may outweigh a possible inhibitory effect on the phagocytic activity of the macrophages. Further research using the zebrafish model may shed light on a possible interplay between these effects, since the *Mm* infection model has been shown to have excellent translational value for human TB, including the effects of GC treatment (32, 40).

In conclusion, our *in vivo* study on the effect of GC treatment in the zebrafish *Mm* infection model shows that GCs, through activation of Gr, inhibit the phagocytic activity of macrophages, and results in more extracellular bacterial growth and a higher infection level. These results may explain why clinically prolonged GC treatment is associated with an increased risk of TB and other bacterial infections.

## Data Availability Statement

Upon request, the raw data supporting the conclusions of this article will be made available by the authors without undue reservation.

## Author Contributions

Conceptualization: AM and MS. Methodology: YX, JX, AM, and MS. Validation: YX and MS. Formal analysis: YX and JX. Investigation: YX and JX. Resources: MS. Data curation: YX and MS. Writing - original draft: YX and MS. Writing - review and editing: YX, JX, AM, and MS. Supervision: AM and MS. Project administration: MS. Funding acquisition: YX. All authors contributed to the article and approved the submitted version.

## Funding

YX and JX were funded by a grant from the China Scholarship Council (CSC).

## Conflict of Interest

The authors declare that the research was conducted in the absence of any commercial or financial relationships that could be construed as a potential conflict of interest.
