# Supplementary material for: Glucocorticoid-Induced Exacerbation of Mycobacterial Infection Is Associated With a Reduced Phagocytic Capacity of Macrophages
Source: Front Immunol. 2021 May 11;12:618569. doi: 10.3389/fimmu.2021.618569 (PMC8148013; doi:10.3389/fimmu.2021.618569)
Supplement: Supplementary file 1 [file Table_1.docx]

**Supplementary material**

**Supplementary Table 1. Sequences of Primers used in qPCR reactions.**

| **Gene name** | **Gene accession** | **Sequence (5'-3')** |
| --- | --- | --- |
| *ppiab* | ENSDARG00000103994 | **Fw:** CATCCACAACCTTCCCGAACAC |
|  |  | **Rv:** ACACTGAAACACGGAGGCAAAG |
| *tnfa* | ENSDARG00000009511 | **Fw:** ACCAGGCCTTTTCTTCAGGT |
|  |  | **Rv:** TTTGCCTCCGTAGGATTCAG |
| *sparcl1* | ENSDARG00000074989 | **Fw:** AACGAGGCTGAGAGCAAGGA |
|  |  | **Rv:** GCTGTGGCTGTGGGGATTAC |
| *uchl1* | ENSDARG00000026871 | **Fw:** GGCCAACAACCAGGACAGTA |
|  |  | **Rv:** CCTCATCAGCAACAGCATCA |
| *ube2v1* | ENSDARG00000041875 | **Fw:** GGTCAGAAGGGTGTTGGAGA |
|  |  | **Rv:** TCGTCACAAAACGGACAAAA |
| *marcksa* | ENSDARG00000004049 | **Fw:** ATGAAGCCAAGACGGATGGA |
|  |  | **Rv:** GCCACTGCTGGCTCAGTTTT |
| *marcksb* | ENSDARG00000008803 | **Fw:** CAAGCTGAGCGGTTTCTCCT |
|  |  | **Rv:** CCTCGGGTTCAGCATCTTTC |
| *bsg* | ENSDARG00000019881 | **Fw:** ATCAAGAGCACCCCCAACAA |
|  |  | **Rv:** CACTGGTGCCTTGACACTGG |
| *tubb5* | ENSDARG00000037997 | **Fw:** CAAACTCACCACCCCCACAT |
|  |  | **Rv:** GCTTGTCAGAGGAGCGAAGC |
